# An Actinobacterium Strain From Soil of Cerrado Promotes Phosphorus Solubilization and Plant Growth in Soybean Plants

**DOI:** 10.3389/fbioe.2021.579906

**Published:** 2021-04-22

**Authors:** Harold Alexander Vargas Hoyos, Josiane Barros Chiaramonte, Ana Gabriele Barbosa-Casteliani, Jorge Fernandez Morais, Juan Esteban Perez-Jaramillo, Suikinai Nobre Santos, Sonia Claudia Nascimento Queiroz, Itamar Soares Melo

**Affiliations:** ^1^Program for the Study and Control of Tropical Diseases—PECET, School of Medicine, University of Antioquia, Medellín, Colombia; ^2^Laboratory of Environmental Microbiology, Embrapa Environment, Jaguariúna, Brazil

**Keywords:** Brazilian biodiversity, wheat rhizosphere, *Streptomyces rishiriensis*, gluconic acid, P-solubilizing potential, plant growth promotion

## Abstract

The huge biological diversity of the Brazilian Cerrado is an important source of economically interesting microbial agents. The phylum Actinobacteria plays an important role in nutrient cycling, potentially improving their availability to plants. In this study, we isolated an actinobacteria (strain 3AS4) from wheat rhizospheres of crops cultivated in the Cerrado biome. Strain 3AS4 was identified as belonging to the genus *Streptomyces* and had phosphorus mobilization ability, mineralizing approximately 410 μg ml^–1^ from phytate, 300 μg ml^–1^ from calcium phosphate, and 200 μg ml^–1^ from rock phosphate. The analysis of the actinobacteria crude extract by spectrometric techniques revealed the presence of gluconic and 2-ketogluconic acid, and a greenhouse experiment was carried out to evaluate its plant growth promotion activity in soybean. Soil in its natural condition (with no phosphorus addition), 40 kg ha^–1^ rock phosphate from Bayovar (RP) added to soil, and triple super phosphate (SPT) added to soil were used. Significant differences in plant height were observed at 6 weeks when the plants were inoculated with the 3AS4 strain. The growth of inoculated plants in natural condition was promoted in 17% compared with the RP and SPT non-inoculated conditions, suggesting that inoculation can enable plants to grow with lower chemical P fertilizers. In the plants that were inoculated with the 3AS4 strain in the RP condition, the plant height increased by approximately 80% and the shoot:root ratio was approximately 30% higher compared to control conditions (non-inoculated plants in natural conditions). 3AS4 has P-solubilizing potential and can be exploited as an inoculant for soybean cultivation. These results suggest that this actinobacterium is a valuable resource for sustainable agriculture and will allow the reduction of phosphate fertilization in the future.

## Introduction

The Cerrado (Brazilian Savanna) biome is the second largest Brazilian biome and occupies an area of approximately 2 million km^2^, which is equivalent to up to 20% of the national territory (Bonanomi et al., 2019). Although it is a hostile environment, this biome has a great wealth of species, genetic resources, and ecosystems (Hidasi-Neto et al., 2019). It is a dry and hot environment, but represents a great source of biodiversity; conversely, it has little global recognition compared with the Amazon basin (Marris, 2005; Fonseca and Venticinque, 2018). Several studies have already investigated this biome as a source of raw materials for drug discovery; potential antifungal, antitumor, and antibiotic agents have been identified from plant and microorganismal origin (Quirino et al., 2009; Calixto Júnior et al., 2016; Souza et al., 2016; de Araujo et al., 2018; Genuário et al., 2019). Owing to the favorable resources of the Cerrado biome, such as the flat topography and appropriate soil depth, amount of rainfall, and light intensity (de Pontes et al., 2017) and despite the low pH, low phosphorus (P) content, and high P fixation capacity (Hinsinger, 2001), the Midwest region of Brazil widely cultivates soybean (*Glycine max* L. Merrill). Moreover since 1970, soybean cultivation has been increasing in the Cerrado, requiring more chemical fertilizer (Roy et al., 2016).

Soybean is one of the most important crops in Brazil (Figueiredo, 2016; Zortea et al., 2018); in the 2018/2019 period, the overall production was 120.3 million tons/year, with a crop yield of 3.833 kg/h (Conab Companhia Nacional de Abastecimento, 2019). It is important owing to its high content (approximately 40%) of vegetable protein (Xing et al., 2018), which constitutes one of the main raw materials for the food industry. A recent study by Da Costa Leite et al. (2018) established that the maximum P efficiency in a soybean production area in the Cerrado, in the state of Bahia, under the no-tillage system (with subsoiling) is 172 kg ha^–1^. Despite this, in Brazil, the input of phosphate fertilizers is high. Withers et al. (2018) showed that P is applied at 25 kg ha^–1^ in the state of Paraná, 35 kg ha^–1^ in the state of Goias, and 50 kg ha^–1^ in the Matopiba Region; 4.6 tg of P will be applied in Brazil by 2050. The frequent input of phosphate into soils contributes to a build-up of legacy P, which will be of great importance during a P crisis (Kochian, 2012; Withers et al., 2018). The mobilization of soil phosphate by microorganisms has been explored as an alternative to increased phosphate fertilizer application (Khan et al., 2017; Sathya et al., 2017) and is an important tool for exploring legacy P in soils. Microorganisms that belong to the phylum Actinobacteria produce biologically active compounds via complex biosynthetic mechanisms, which enable them to adapt to different ecosystems (Singh et al., 2018), and may act as plant growth promotion molecules (El-Tarabily et al., 2008; Saif et al., 2014). The role of Actinobacteria in promoting plant growth owing to the solubilization of phosphates in the soil has been explored (Palaniyandi et al., 2013; Vurukonda et al., 2018). Microorganisms in this phylum are involved in several biological processes, producing a wide range of bioactive compounds, such as chitinases (Lacombe-Harvey et al., 2018) and cellulase (Bettache et al., 2018). Thus, the aim of the present study was to evaluate the P mobilization potential of Actinobacteria from the Cerrado biome, investigate the possible mechanisms of action, and verify the potential of Actinobacteria to promote the growth of soybean plants.

## Results

### Isolation and Purification of the Isolates

Sixteen strains belonging to the phylum of actinobacteria were isolated from the rhizospheres of wheat. Screening to verify P mobilization ability showed that three isolates were able to mineralize phytate (3AS4, 2BS5, and 3BS2) (Supplementary Table 1); isolate 3AS4 also had the ability to solubilize calcium and rock phosphate (Supplementary Figures 1A,B) and was selected for phenotypic description, molecular identification, and testing under greenhouse conditions.

### Genotypic and Phenotypic Characterization of Strain 3AS4

**FIGURE 1:**
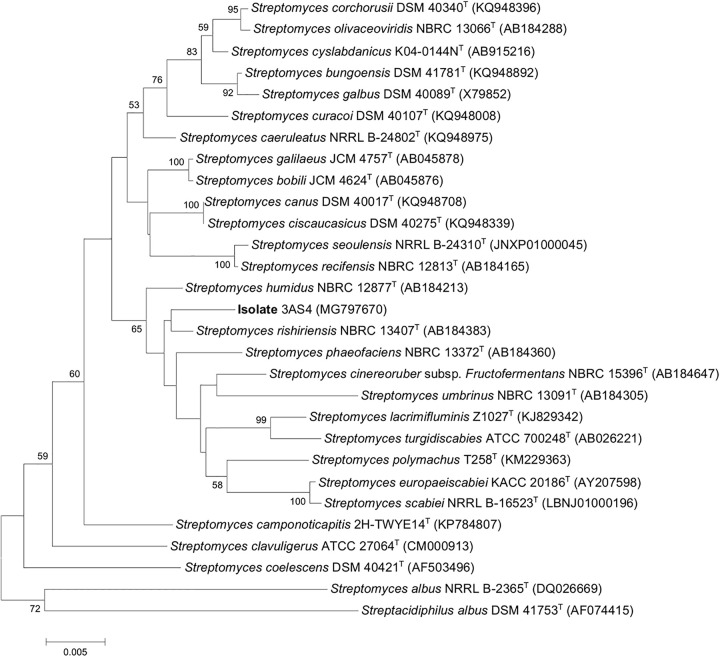
Neighbor-joining phylogenetic tree based on nearly complete 16S rRNA gene sequences showing relationships between 3AS4 strain and closely related type strains of *Streptomyces* species.

The phylogenetic tree assembled from the 16S rRNA sequence (1.493 nt) indicated that the isolate belongs to the genus *Streptomyces* and has a 99% of similarity with *Streptomyces rishiriensis* NBRC 13407^*T*^ (Figure 1). With this strain, a defined phylogenetic branch was formed (Figure 1). The accession number for the 3AS4 isolate was MG797670. Isolate 3AS4 had similar morphology and physiology (Supplementary Figures 2, 3) to *S. rishiriensis*, which produces diffusible pigments in the medium, grows slowly in solid media, and does not produce abundant aerial mycelia or spores (Charousová et al., 2015).

Strain 3AS4 grew well on ISP1, 3, and 4 media and moderately on ISP2, 5, 6, and 7 media. In all ISP media, the reverse of the colony was light brown, except for ISP2 (yellowish/white). The substrate mycelium was whitish to light brown, and the aerial mycelium was present in ISP1, 3, and 4 media, but diffusible pigments were observed only in ISP1 and 3 media (Supplementary Figure 3). A branched, whitish substrate mycelium and pale grayish aerial hypha, which divided into straight to flexuous (rectiflexibilis) chains with smooth surfaces and rod-shaped spores, formed on oatmeal agar (ISP3 medium; Supplementary Figure 4). The strain 3AS4 grew at 16–37°C (optimal, 28°C) and pH 5–10 (optimal, 7.0) and was tolerant of 15% NaCl. Additionally, 3AS4 grew with hydrolyzed starch, casein, cellobiose, D-arabinose, D-galactose, D-maltose, glucose, guanine, hypoxanthine, raffinose, rhamnose, mannitol, *myo*-inositol, sucrose, and xylitol but not adenine, D-mannose, and D-ribose. Moreover, it grew in degraded tyrosine and Tween 20 and 80 but not xanthine. The 3AS4 strain showed sensitivity (μg ml^–1^) to rifampicin (10), streptomycin (16), and ampicillin (10) but not erythromycin (15). The strain possessed enzymatic activity against acid phosphatase, alkaline phosphatase, α-glucosidase, β-galactosidase, β-glucosidase, esterase (C4), esterase lipase (C8), leucine arylamidase, valine arylamidase, N-acetyl-β-glucosaminidase, and naphthol-phosphohydrolase.

### Potential for P Mobilization

**FIGURE 2:**
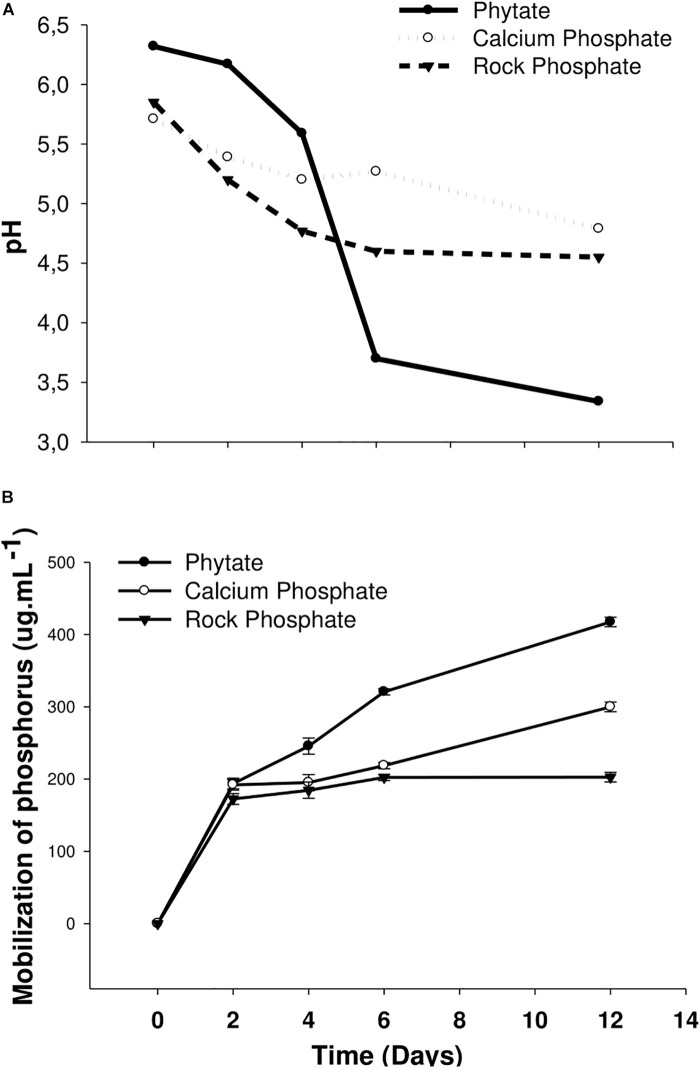
Analysis of the behavior of pH **(A)** and mobilization of phosphorus **(B)** in three different media {Phytate, Calcium Phosphate [Ca_3_(PO_4_)_2_], and Rock Phosphate} inoculated with the 3AS4 strain, grown for 12 days at 28°C under stirring.

The liquid culture in both calcium and rock phosphate media showed a decrease in pH with time (Figure 2A). This indicates that solubilization occurred owing to the liberation of organic acids by microorganisms during siderophore excretion. The major liberation of phosphate occurred after 12 days in both cases; moreover, the largest amount of phosphate was liberated from phytate (Figure 2B). Phosphate release by the 3AS4 isolate (on days 2–12) ranged from 150 to 410 μg ml^–1^ in the phytate medium, 190–300 μg ml^–1^ in the calcium phosphate medium, and 170–200 μg ml^–1^ in the rock phosphate medium.

### Identification of Organic Acids

**FIGURE 3:**
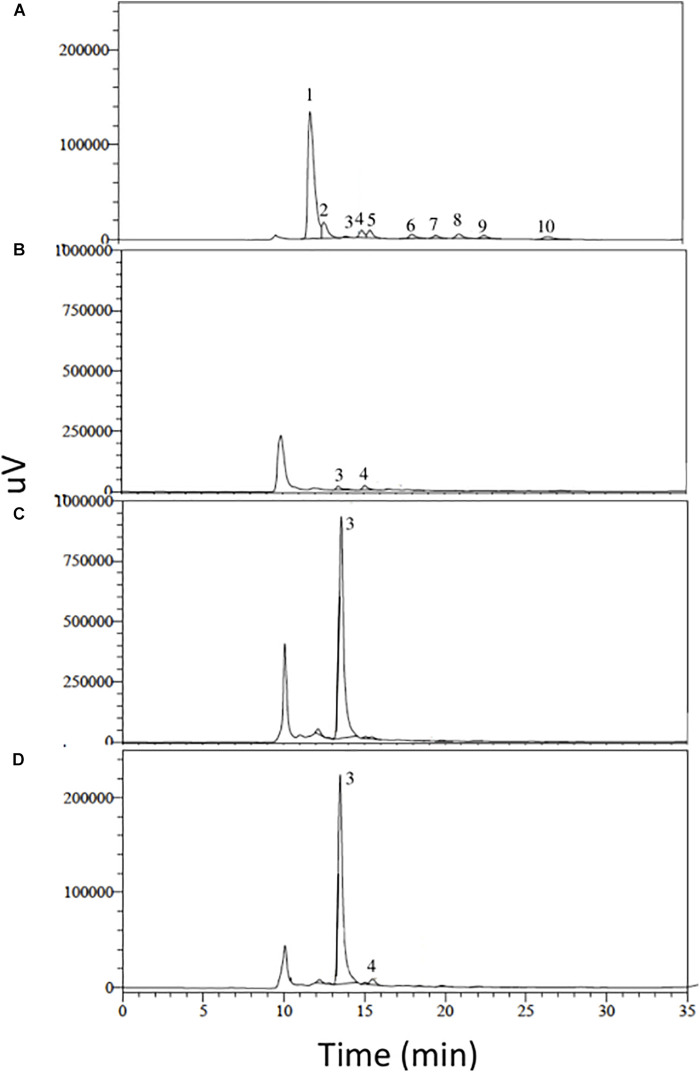
HPLC analysis of organic acids involved in phosphorus solubilization. **(A)** Chromatographic profile of organic acid standards. **(B)** Extract of the 3AS4 strain obtained in phytate medium. **(C)** Extract of the 3AS4 strain obtained in calcium phosphate medium [Ca_3_(PO_4_)_2_]. **(D)** Extract of the 3AS4 strain obtained in Rock Phosphate medium. All extracts were obtained after 12 days of growth in liquid medium under agitation (140 rpm) at 28°C. Numbers represent the different organic acids used, 1: oxalic acid, 2: citric acid, 3: gluconic acid, 4: 2-ketogluconic acid, 5: malonic acid, 6: succinic acid, 7: lactic acid, 8: formic acid, 9: malic acid, 10: propionic acid.

As shown by high-performance liquid chromatography (HPLC), the gluconic acid standard and the aqueous extract of 3AS4 had similar chromatographic profiles after 12 days of incubation (Figure 3).

### Cellulolytic Enzymatic Complex Activity

The 3AS4 strain showed cellulase activity, 2BS5 showed chitinase activity, and 3BS2 showed cellulase, glucanase, and xylanase activities (Supplementary Table 1). All three isolates could inhibit the mycelial growth of *Sclerotinia sclerotiorum in vitro* by 48, 35, and 59%, respectively. Of the three pre-selected isolates, only the 3AS4 strain produced indole acetic acid (Supplementary Table 1), which is associated with the promotion of plant growth (Fialho de Oliveira et al., 2010). Actinobacteria isolates showed a positive association with some other strains of microorganisms (data not shown) isolated from the soybean culture, such as *Bradyrhizobium* sp., *Rhizobium* sp., and *Azospirillum* sp.

### Greenhouse Experiment

**FIGURE 4:**
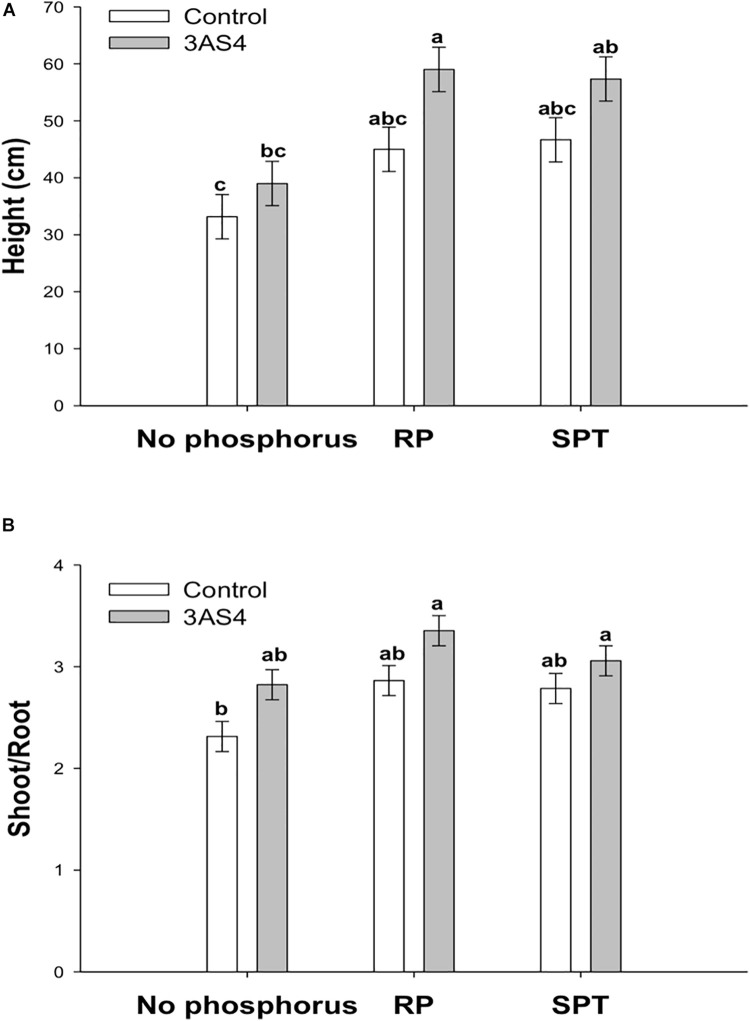
Comparison of the height **(A)** and shoot:root ratio **(B)** of the soybean plants inoculated with the 3AS4 strain with two different sources of phosphorus. Rock phosphate (RP) and Single super phosphate (SPT) at 40 kg ha^–1^. The histograms with the same letter at each case are not significantly different at *P* < 0.05 (Tukey’s test).

**FIGURE 5:**
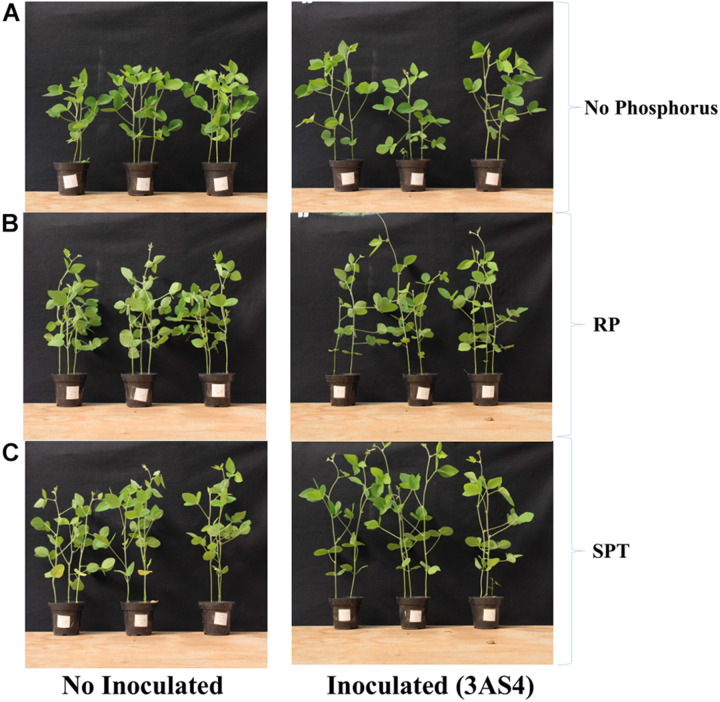
Evaluation of the height of the soybean plants 6 weeks after inoculated with the 3AS4 strain with two different sources of phosphorus. **(A)** Soybean plants without inoculation of 3AS4 (left), inoculated with 3AS4 isolate and no additional phosphorus. **(B)** Soybean plants without inoculation of 3AS4 (left), inoculated with 3AS4 isolate and Rock phosphate (RP) at 40 kg ha^–1^. **(C)** Soybean plants without inoculation of 3AS4 (left), inoculated with 3AS4 isolate and Single super phosphate (SPT) at 40 kg ha^–1^.

The 3SA4 strain was efficient at P solubilization and mineralization *in vitro*. To verify the performance in colonizing the rhizosphere and promoting plant growth, a greenhouse experiment was carried out. 3AS4 inoculation into soil without the addition of P promoted a 17% increase in the height of plants compared with control plants grown under the same conditions (Figures 4A, 5).

When the 3AS4 strain was inoculated with an insoluble P source (RP), shoot growth was almost 80% greater than that in plants grown under P-limiting conditions (Figure 4A). The shoot:root ratio was approximately 30% higher when rock phosphate was added with the isolate than when only phosphate was used (Figure 4B).

## Discussion

### Genotypic Characterization

The morphological and genetic characteristics of the 3AS4 strain indicate that it is part of the *Streptomyces* genus. The 16S rRNA sequence data supported the assignment of the isolate 3AS4 to *S. rishiriensis* (NBRC 13407^*T*^) with 99.03% similarity. *S. rishiriensis* produces antibiotics (Matsumoto et al., 1999; Wang et al., 2000; Charousová et al., 2015); however, to the best of our knowledge, this is the first report of this species as a growth stimulant in soybean plants.

### Potential of Phosphorus Mobilization

Previous studies have reported high numbers of Actinobacteria in Cerrado soils (Nascimento et al., 2005; Suela Silva et al., 2013; de Sales et al., 2017; Vieira et al., 2018; Vargas Hoyos et al., 2019). These soils are acidic and have a low availability of macronutrients (nitrogen, P, potassium, calcium, magnesium, and sulfur) and micronutrients (boron, copper, molybdenum, and zinc) (Cruz Ruggiero et al., 2002; Oliveira and Marquis, 2002; Genuário et al., 2019). Moreover, these soils usually show high aluminum saturation and P fixation potential (Lopes, 1996; Junio de Jesús Lacerda et al., 2015; Mariussi et al., 2019). The presence of P-mobilizing bacteria in the rhizosphere might allow a higher availability of P to plants in a beneficial association, where bacteria are provided with nutrients and niche to thrive in the rhizosphere (Oliveira et al., 2009; Estrada-Bonilla et al., 2017; Zheng et al., 2019).

Therefore, this microhabitat should be explored for potential inoculants. Previous studies have reported the liberation of phosphate by Actinobacteria, especially by *Streptomyces* species. Hamdali et al. (2008) reported the capacity of several strains isolated from three Moroccan phosphate mining centers to solubilize phosphate in a synthetic minimum medium supplemented with rock phosphate; two had values of 21.43 and 29.67 μg L^–1^ after a 5 day incubation at 28°C. Oliveira et al. (2009) described six strains that belong to the *Streptomyces* genus isolated from Cerrado rhizosphere maize plants; *S. platensis*, *S. tumescens*, *S. chartreusis*, *S. griseochromogenes*, *S. collinus*, and *S. avermitillis* are capable of solubilizing 68, 43.1, 23.5, 4.1, 1.8, and 3.7 mg phosphate L^–1^ tricalcium phosphate, respectively. In the same study, two *Arthrobacter* strains solubilized 28 and 30.5 mg phosphate L^–1^ sodium phytate after a 10 day incubation at 27°C. Wang et al. (2018) describes a strain of *S. griseoplanus* isolated from the maize rhizosphere that solubilized 284.80 μg phosphate ml^–1^ tricalcium phosphate after a 20 day incubation at 28°C. Similarly, Solans et al. (2019) evaluated five actinobacterial strains, two of which, belonging to the genus *Streptomyces*, were the most efficient solubilizers of Ca_3_PO_4_ of 2.27 and 2.149 mg L^–1^ (PO4^–2^) g^–1^, respectively, after 14 days at 28°C.

The results obtained in this study showed that, after 3AS4 inoculation, 410 μg ml^–1^ phosphate was released from phytate, 300 μg ml^–1^ was released from calcium phosphate, and 200 μg. ml^–1^ was released from rock phosphate *in vitro*. It is important to highlight that the time required for P mobilization was relatively short (12 days) and the temperature required was moderate (28°C).

Soils vary greatly in pH and other characteristics; therefore, there is no phosphate compound most suitable to screen the most efficient mobilizing microorganisms. Therefore, more than one source of phosphate should be used to screen organic compounds and rock phosphate (Bashan et al., 2013). The most frequently evaluated source of P is (tri-)calcium phosphate. The results of this study showed the ability of *S. rishiriensis* to solubilize both organic and inorganic P, demonstrating the great potential of the 3AS4 strain for P solubilization. Furthermore, positive results for the acid and alkaline phosphatase enzymes suggest that more than one mobilization process could be carried out by this isolate.

### Identification of Organic Acids

Microorganisms that mobilize P at low pH levels, often owing to the production of organic acids, have been widely documented. Different genera of actinobacteria, such as *Micromonospora* and *Streptomyces*, have been reported to solubilize phosphate through siderophore production (Hamdali et al., 2008, 2010; Gopalakrishnan et al., 2014). Moreover, genera such as *Arthrobacter* and *Actinomadura* are able to produce organic acids, such as lactic and citric acids (Sharma et al., 2013). However, gluconic acid is the most commonly reported and most efficient solubilizing agent (Alori et al., 2017).

Recent studies have demonstrated the presence of organic acids in *Streptomyces* isolates with the capacity to stimulate growth in plants through the release of free phosphate. Jog et al. (2014) reported two *Streptomyces* strains capable of producing gluconic and malic acids. Similarly, Farhat et al. (2015) evaluated 128 isolates of actinobacteria and selected two, belonging to the genus *Streptomyces*, that possessed a remarkable capacity to produce gluconic acid. Prieto-Correal et al. (2015) evaluated 15 isolates of *Streptomyces*, 14 of which were able to produce organic acids in both tricalcium phosphate and aluminum phosphate media, most frequently citric, tartaric, and gluconic acids. Solans et al. (2019) evaluated five *Streptomyces* isolates, all of which were able to produce organic acids in both calcium phosphate and aluminum phosphate media, most frequently gluconic, oxalic, citric, and malic acids. In this study, we identified a *S. rishiriensis* isolate with the ability to solubilize P and identified that both gluconic and 2-ketogluconic acids were involved in the process. In general, the majority of P-solubilizing microorganisms produce gluconic acid but not 2-ketogluconic acid (Kumar et al., 2013). However, there is a common organic acid profile in the isolates of *Streptomyces* sp., in which gluconic acid is often found, whereas 2-ketogluconic acid is less frequently reported. It would be useful to search for an operon associated with the production of 2-ketogluconic acid, as is the case for gluconate dehydrogenase (*gad*), to enhance the production of this organic acid, as has been successfully identified for some gram negative bacterial genera (Kumar et al., 2013).

### Cellulolytic Enzymatic Complex Activities

Enzymatic activity might be one of the main mechanisms of the control of soil-borne pathogens by actinobacteria (El-Tarabily et al., 2009; Kaur et al., 2013; Swiontek Brzezinska et al., 2013), such as *S. sclerotiorum*, which is responsible for the loss of 20–30% and, in some cases, at least 70% of soybean crops in Brazil (de Oliveira et al., 2019; Meyer et al., 2019). It is estimated that approximately 28% of the total area of production is infected, compromising almost 10 million ha (Meyer et al., 2019).

In addition to the ability to mobilize phosphate, microorganisms usually show potential to produce indole acetic acid (AIA) and siderophores, which also promote plant growth (Fialho de Oliveira et al., 2010; Shrivastava et al., 2017). The selected isolate in this study also showed the capacity to produce AIA; this result is consistent with those of previous studies that demonstrated the liberation of this hormone by *Streptomyces* sp. with plant growth-promoting activity (Abd-Alla et al., 2013; Myo et al., 2019; Chukwuneme et al., 2020). The siderophore is also responsible for the mobilization of phosphate by the formation of stable complexes with P from aluminum, iron, and calcium compounds (Hamdali et al., 2008; Soumare et al., 2020).

### Greenhouse Experiment

Soybean crops require a lot of nutrients, especially nitrogen and P. Since 1960, when soybeans began to be produced on a commercial scale in Brazil, measures were adopted, emphasizing biological nitrogen fixation (Hungria and Vargas, 2000). Currently, the soybean crop obtains the greatest proportion of nitrogen from biological fixation (Ambrosini et al., 2019). This was achieved by the selection of rhizobia for the formulation of inoculants and selection of plants predisposed to nodulation. The second nutrient limiting soybean production is P. In Brazil, soybean cultivation is responsible for more of the input of phosphate fertilizers to the soil than any other crop (Withers et al., 2018). P is strongly attached to colloids in tropical acidic soils, mainly by iron and aluminum; thus, less than 0.1% of soil P is in its soluble form (Silva et al., 2019). As with nitrogen, the application of inoculants based on microorganisms capable of releasing soil-bound P has great potential to increase the availability of nutrients for agriculture (Kumar et al., 2018; Kalayu, 2019; Sammauria et al., 2020).

One strategy to reduce P fixation in the soil is the use of less soluble P sources. The application of rock phosphates presents several advantages compared with the application of other phosphate fertilizers (Van Straaten, 2006; Hellal et al., 2019). Because they do not undergo the same chemical process as the production of conventional fertilizers, phosphate rocks are usually cheaper and have lower environmental impact processes (Zapata and Roy, 2004; Schröder et al., 2011). Additionally, owing to their variable and complex chemical composition, they are sources of several nutrients in addition to P. The slow release of nutrients from the rock to the soil supplies the nutritional requirements of agricultural crops and reduces the rate of P fixation (Zapata and Roy, 2004; Mishra and Nayak, 2020). However, a major problem with the use of rock phosphates as a source of nutrients is that in the initial years, production tends to be lower than that with soluble phosphates (Hellal et al., 2019).

Sustainable agriculture is being promoted by practices such as soil management, the genetic engineering of plants, and the application of inoculants to increase nutrient intake (Nimnoi et al., 2014; de Sousa and Olivares, 2016). The solubilization, mobilization, assimilation, and use of P have been the focus of several studies in recent years (Tian et al., 2012; Nesme et al., 2014; Kalayu, 2019). The application of inoculants to the soil has been shown to be sustainable and beneficial to the environment owing to the reduction in the need for P fertilizer application (Kaur et al., 2013; Sharma et al., 2013). The response of the plant to inoculated microorganisms is usually related to complex interactions with the soil and rhizospheric communities adapted to the plant (Jones and Oburger, 2011; Ahkami et al., 2017; Vurukonda et al., 2018).

In this study, isolate 3AS4 played a significant role in the promotion of soybean growth; it increased the height of plants by 17% (Figures 4A, 5), which agrees with Henri (2014), who indicated that the addition of microorganisms that solubilize P can increase plant growth by between 10 and 15%.

When the 3AS4 strain was inoculated into a pot with an insoluble phosphorus source (RP), soybean plants presented an almost 80% increase in shoot growth and 30% increase in the shoot:root ratio (Figures 4B, 5). These results are complementary to those already reported by Fatmawati et al. (2019), who described six isolates of actinobacteria that stimulated plant growth *in vitro* in soybean by exerting significant effects on root elongation, the number of lateral roots, and root dry weight. Mba (1994) described an increase in soybean production under field conditions after the co-inoculation of rock phosphate and two isolates of actinobacteria (by 43% and 17%, respectively). Thus, the synergistic effect of 3AS4 and P can be explained by the release of hormones that promote plant growth (Gopalakrishnan et al., 2014; Htwe et al., 2018). We showed that 3AS4 also releases IAA (Supplementary Table 1), and as previously suggested, Actinobacteria that produce this phytohormone aid plant growth (Sathya et al., 2017). There are several reports of actinobacteria as promoters of plant growth in different species; these emphasize the use of these microorganisms and their metabolic products to increase production and control diseases in grain legumes (Sathya et al., 2017) and existing commercial techniques that are routinely employed in vegetable farming (Chaurasia et al., 2018).

## Conclusion

Brazil has biomes with distinct environmental characteristics, which can serve as a source of microorganisms with significant biotechnological potential. The 3AS4 isolate showed important P-solubilizing potential and can be exploited as an inoculant for soybean cultivation. Moreover, inoculation of this microorganism with rock phosphate may be a promising strategy to promote crop growth, minimizing losses related to fertilization with soluble P sources and reducing P fertilizer use in soils. There is interest to discover new microorganisms that can facilitate productive processes in agriculture. In this sense, the phylum Actinobacteria requires additional focus, as the species in this phylum are versatile and have the capacity to survive in different environments. To effectively utilize the process of plant nutrient acquisition, efficient mechanisms in the cycling of nutrients from the soil must be identified; then, biofertilizers obtained from soils can be developed.

In conclusion, the 3AS4 strain was isolated, characterized, and shown to be a strain of *S. rishiriensis* with outstanding biological capabilities, among them, the solubilization of organic and inorganic P. Thus, this strain is an appropriate candidate as an alternative tool for crop fertilization, to be combined with chemical fertilizers, which will result in less consumption of chemical fertilizers and greater availability of phosphorus for soybean plants.

## Materials and Methods

### Isolation and Purification

Two sampling sites were performed to obtain the isolates. The first one occurred in Palmital (S 22∘ 47′ 30″; W 50∘ 12′ 18″) São Paulo State, and Planaltina (S 15∘ 36′; W 47∘ 42′) Brasília, DF. The sites had historic of rotation management between soybean crop and wheat crop. The collected material consisted in rhizosphere of the wheat crop. The soil strongly adhered to the wheat roots were collected in plastic bags. The samples were diluted in sterile saline solution (NaCl 0.85%), submitted to agitation for 10 min and sonicated during 20 s, to release the attached microorganisms from soil particles (Shirling and Gottlieb, 1966). Serial dilutions were performed and a subsample of 0.1 ml at 10^–4^ was cultured in Glucose Yeast Agar (GYA) (Gordon and Mihm, 1962). After the isolation, the Actinobacteria were cultivated in international *Streptomyces* project Media 2, ISP2, to confirm the purity and stored at 4°C until downstream analysis.

### Genotypic and Phenotypic Characterization of Strain 3AS4

DNA extraction was made using Ultra Clean^®^ Microbial DNA Isolation, MO Bio (Calif, United States), according to the manufacturer’s instructions. 16S rRNA gene was amplified with primers: 27F (5′-AGA GTT TGA TCM TGG CTC AG-3′) and 1492R (5′-TAC GGY TAC CTT GTT ACG ACT T-3′). Amplification products were purified using Promega Wizard SV gel and PCR clean-up system. The sequencing reaction was performed with primers 27F, 704F (5′-AGA TTT TCC GAC GGC AGG TT-3′), 1114R (5′-GGG TTG CGC TCG TTG C-3′), and 1492R, using BIGDYE protocol (Applied biosystems, Foster City, Calif.) in 3500 Genomic Analyzer Sequencer (Applied Biosystems). The sequences were assembled using the CLC Software^1^ and compared to strains with the Ez-Taxón e-server (Kim et al., 2012). The alignment of sequences was performed with *Clustal W* (Thompson et al., 1994) and phylogenetic trees were inferred by neighbor-joining method (Saitou and Nei, 1987) in MEGA 7 (Kumar et al., 2016).

The growth cultural characteristics were assessed using standard ISP media, after inoculation at 28∘C for 3 weeks. Characterization of the temperature growth, pH, NaCl tolerance, and antibiotic sensitivity was conducted in Glucose Yeast Extract Agar (GYEA) (Shirling and Gottlieb, 1966). The assimilation of carbon sources and degradation of compounds were checked using the basal culture medium (Sigma) and GYEA, respectively (Gordon and Mihm, 1962). Morphological observations of spore arrangement and ornamentation were conducted by scanning electron microscopy of oatmeal agar (ISP-3) cultures incubated at 28∘C for 3 weeks, using the gold-coated dehydrated samples that were made as described by O’Donnell et al. (1993). Enzymatic activities were determined using API^®^ZYM strips (BioMérieux), according to the manufacturer’s instructions. Dual culture assay was performed against *S. sclerotiorum* as described by Cheng et al. (2014).

### Phosphorus Mobilization Activity

The actinobacteria isolates were submitted to assays for assessing the potential for mobilization and mineralization of different sources of phosphates such as phytate and rock phosphates using the reference medium NBRIP (National Botanical Research Institute’s Phosphate) (Nautiyal, 1999) and modified NBRIP (Gadagi and Sa, 2002; Singh et al., 2013).

To quantify the amount of phosphate liberated by actinobacteria from the media, 100 μl of 1 × 10^8^ cells of the strain 3AS4 (OD_560_ = 0.5; Marra et al., 2011) were inoculated in liquid Calcium Phosphate, Phytate, and Rock Phosphate media and incubated at 28^*o*^C at 140 rpm; pH measurements and subsamples were taken after 0, 2, 4, 6, and 12 days. The samples were centrifuged at 15,000g for 5 min, and the amount of phosphorus in the supernatant was quantified by the phosphomolybdenum method (Murphy and Riley, 1962). The aqueous phase was filtered on a Millipore^®^ 0.02 μm membrane and applied in high-performance liquid chromatography (HPLC) to verify and identify the production of organic acids. For the separation, we used an Aminex HPX 87H-300 × 7.8 mm column (125-0140 Bio Rad), mobile phase Water:Sulfuric acid 0.005 M, with a flow rate of 0.4 ml min^–1^, an oven temperature of 50°C, and 20 μl injection volume. Samples were analyzed at the 210 nm length. Analytical curves were performed with the organic acid standards: Oxalic, Citric, Gluconic, 2-ketogluconic, Malonic, Succinic, Lactic, Formic, Malic, and Propionic (Sigma-Aldrich).

### Evaluation of Cellulolytic Enzymatic Complex Activity

The isolates were evaluated for cellulase, chitinase, glucanase, and xylanase activity in SMM (Supplemented Minimal Media) with the addition of 1% of specific substrate: CMC (Sigma C5013), Chitin (Sigma C7170), Laminarin (Sigma L9634), and Xilan (Sigma X4252), respectively. The presence of hydrolysis halo around the isolate confirmed the enzymatic activity, which was classified as follows: 1–10 mm as low (+), 11–20 mm as medium (++), and >21 mm as high (+++) enzymatic activity. Qualitative screening of IAA in vitro production was determined by the previous methodology of Kavamura et al. (2013). Compatibility assays were performed between Actinobacteria and beneficial soybean microorganisms, namely, *Bradyrhizobium* sp., *Rhizobium* sp., and *Azospirillum* sp. (Kavamura et al., 2013).

### Greenhouse Experiment

Soil was obtained at Embrapa Environment experimental field, Jaguariúna SP; this soil does not have a history of previous fertilization practices and is considered phosphorus (P)-poor soil. Eighteen pots, each containing 400 g of soil, were cultivated with three soybean seed var. Potencia 99 (Embrapa-Soja) under glasshouse conditions (i.e., ±35°C, natural luminosity ±11 h light, 70% field capacity). The potential effect of the actinobacteria was evaluated comparing the inoculated and non-inoculated pots in the absence of phosphorus addition and with the addition of two different sources of phosphorus in a total dose of 40 kg ha^–1^ P_2_O_5_, a total of six treatments each with three replicates. The phosphate sources used in this experiment was Triple Superphosphate (TSP) containing 46% of P_2_O_5_ and rock phosphate (RP) containing 31% P_2_O_5_, 4.42% SiO_2_, 0.96% Al_2_O_3_, 0.87% Fe_2_O_3_, 46% CaO, 0.53% MgO, 1.98% Na_2_O, 0.3% K_2_O, 0.01% MnO, and 10.57% LOI. The inoculum was obtained from the filtered biomass of the actinobacteria culture growing in Potato Dextrose (PD; Becton Dickinson) under constant agitation of 135 rpm at 28°C for 7 days. Three milliliters of spore suspension (10^8^ cells) was applied to the pots near each plant. Evaluations occurred after 6 weeks, at the beginning of the flowering stage (R1), considering plant height; after that, shoots and roots were oven dried at 70°C for 72 h to record their dry biomass. Percentage of effect caused by each treatment was calculated according to Henri (2014), considering the negative control and the treatment with no phosphorus addition and no inoculation.

### Statistical Analysis

Data obtained were analyzed by Sigma Plot (Version 10; Systat Software). A one-way ANOVA was used to determine the effects of the treatments, which was assumed to be different when the contrast showed a significance level of *P* ≤ 0.05 using the Tukey test.

## Data Availability Statement

The datasets presented in this study can be found in online repositories. The GenBank accession number for the 16S rRNA sequence of *Streptomyces* sp. 3AS4 is MG797670.

## Author Contributions

HV, JC, SNo, and IS designed the experiments. HV, AB-C, and JF performed antagonistic and enzymatic evaluations. HV and JC performed phosphorus mobilization and greenhouse experiments. HV, JP-J, and SNo performed genetic characterization. HV, JC, SNo, and SNa performed chemical work and data analysis. HV, JC, JP-J, and IS wrote the manuscript with contributions from all authors.

## Conflict of Interest

The authors declare that the research was conducted in the absence of any commercial or financial relationships that could be construed as a potential conflict of interest.

## Footnotes

1 https://www.qiagenbioinformatics.com/
