# Supplementary material for: An Actinobacterium Strain From Soil of Cerrado Promotes Phosphorus Solubilization and Plant Growth in Soybean Plants
Source: Front Bioeng Biotechnol. 2021 Apr 22;9:579906. doi: 10.3389/fbioe.2021.579906 (PMC8100043; doi:10.3389/fbioe.2021.579906)
Supplement: Supplementary file 1 [file Data_Sheet_1.docx]

Supplementary Material

**An Actinobacterium Strain From Soil of Cerrado Promotes Phosphorus Solubilization and Plant Growth in Soybean Plants**

## Supplementary Figures


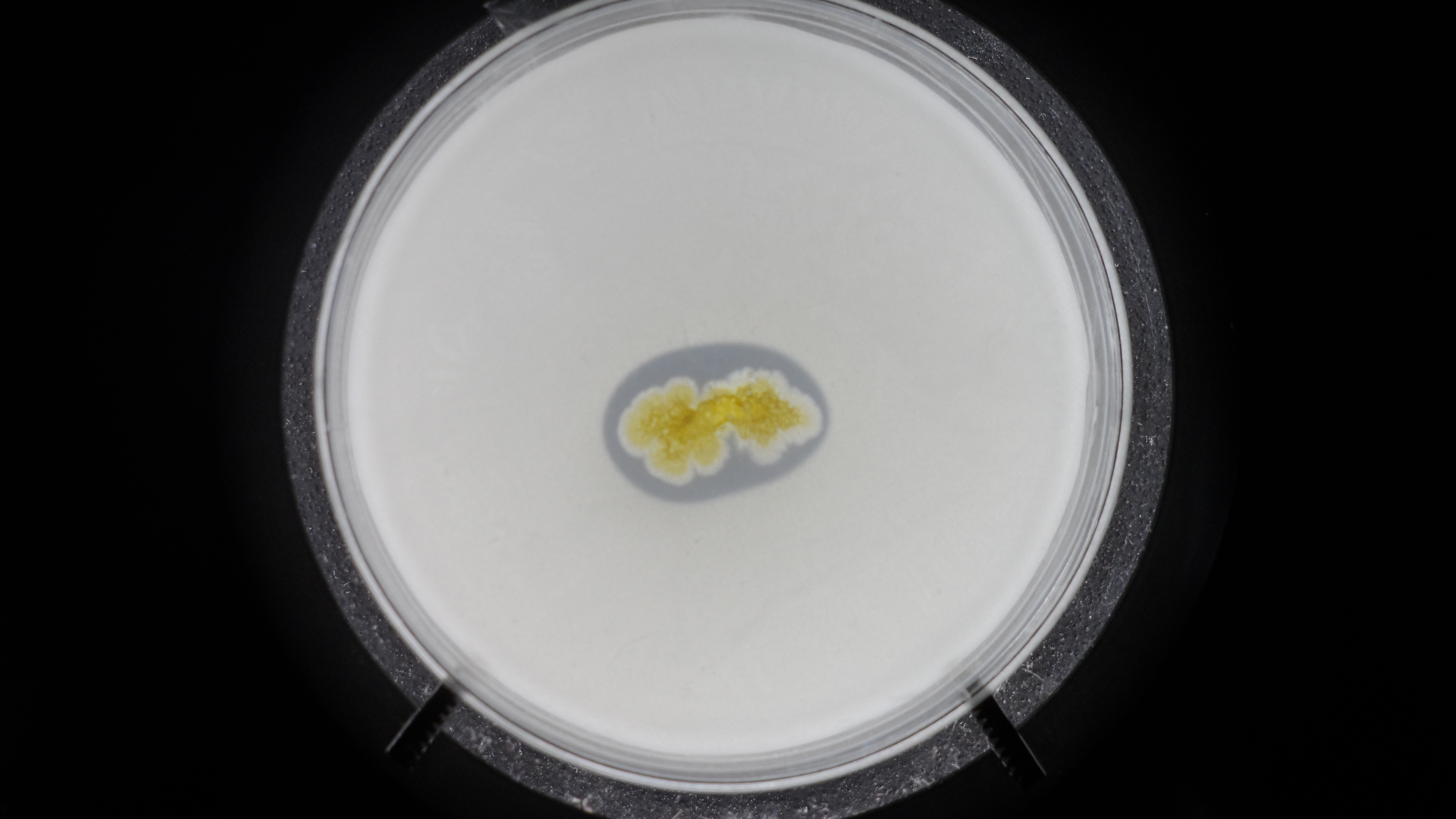

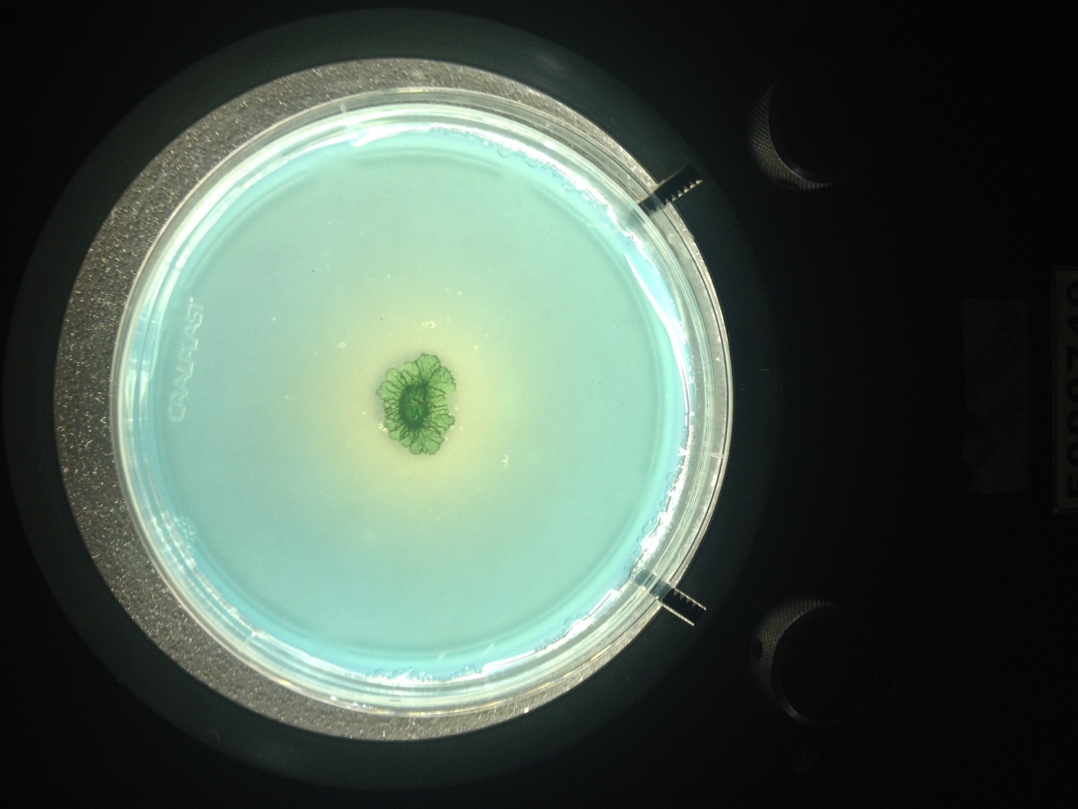


A

B

**Supplementary Figure 1**. Phosphorus hydrolysis by the 3AS4 isolate using two different culture media, grown at 28°C for 2 weeks. (A); NBRIP Medium, (B): Rock Phosphate

| **Phenotypic Characteristics** | **Strain 3AS4** |
| --- | --- |
| **Growth at; pH** |  |
| 3 | - |
| 5 | + |
| 8 | + |
| 10 | + |
| **Temperature** |  |
| 10 | - |
| 16 | + |
| 28 | + |
| 37 | + |
| 40 | - |
| **NaCl tolerance** **(%)** |  |
| 5 | + |
| 10 | + |
| 15 | + |
| **Utilization of** |  |
| Adenine | - |
| Starch | + |
| Casein | + |
| Cellobiose | + |
| D-arabinose | + |
| D-galactose | + |
| D-maltose | + |
| D-mannose | - |
| D-ribose | - |
| Glucose | + |
| Guanine | + |
| Hypoxanthine | + |
| Raffinose | + |
| Rhamnose | + |
| Mannitol | + |
| *myo*-inositol | + |
| Sucrose | + |
| Xylitol | + |
| **Degradation of** |  |
| Tyrosine | + |
| Tween 20 | + |
| Tween 80 | + |
| Xanthine | - |
| **Antibiotic sensitivity (ug.mL^-1^)** |  |
| Rifampicin (10) | + |
| Streptomycin (16) | + |
| Ampicillin (10) | + |
| Erythromycin (15) | - |

Abbreviations: Negative: (-), Positive (+).

**Supplementary Figure 2**. Phenotypic characteristics of strain 3AS4 after incubation at 28°C for 3 weeks.

Abbreviations: Negative: (Neg.)

**Supplementary Figure 3**. Growth and cultural characteristics of strain 3AS4 after incubation at 28°C for 3 weeks.

**A**


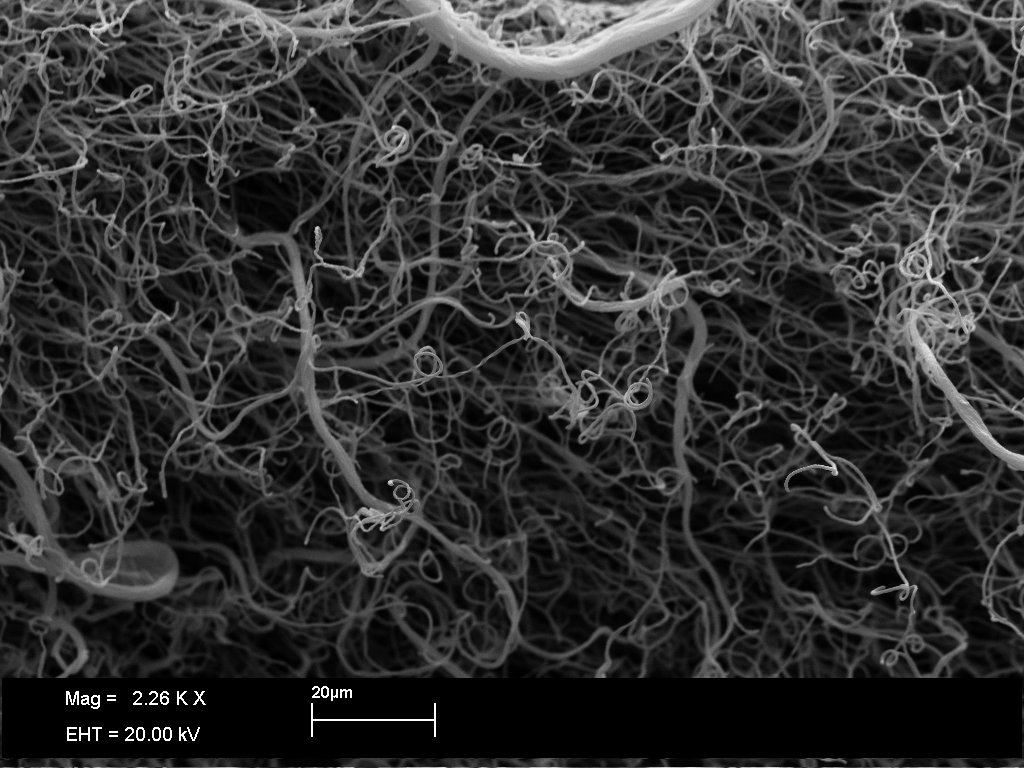


**B**

**
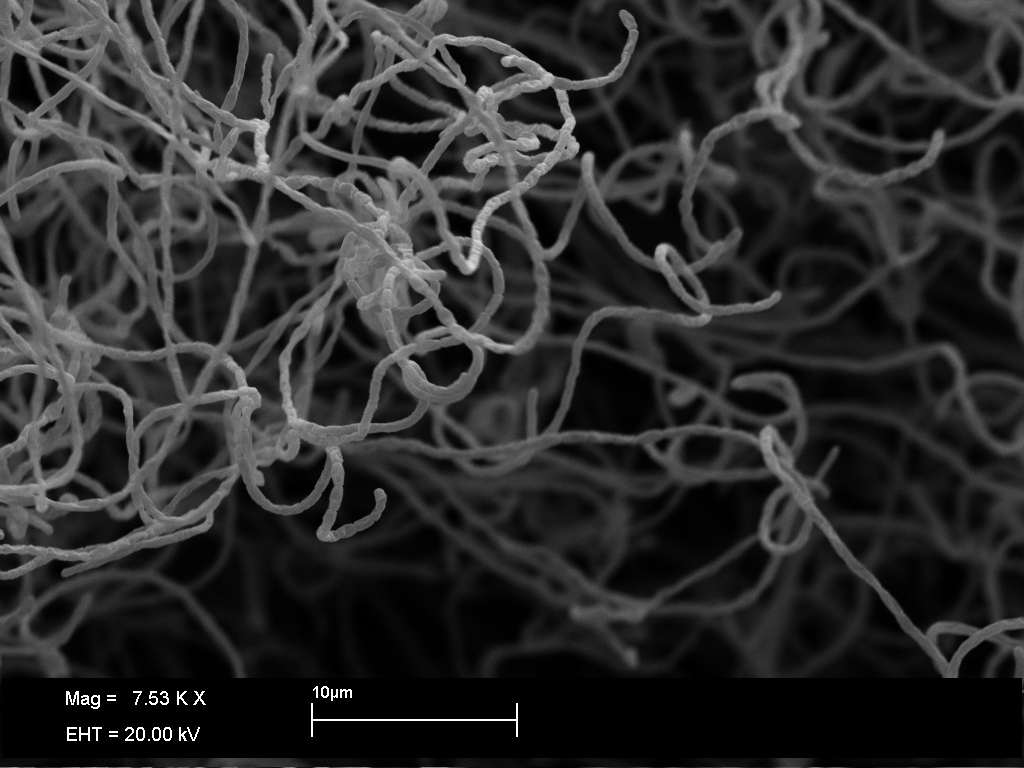
**

**Supplementary Figure 4**. Electron micrograph of strain 3AS4 grown on an oatmeal agar ISP3 at 28°C for 3 weeks. (a) arrangement of the hyphae, Bats 20 μm (b) ornamentation of the spores, Bats 10 μm.

| **Isolates** | **Phosphate Mobilization** | | | **Enzymatic Assay** | | | | **PGPR** |
| --- | --- | --- | --- | --- | --- | --- | --- | --- |
|  | **Ca_3_(PO_4_)_2_** | **Phytate** | **RP** | **Celulase** | **Chitinase** | **Glucanase** | **Xilanase** | **IAA** |
| **3 AS 4** | + | + | + | + | - | - | - | + |
| **2 BS 5** | - | + | - | - | + | - | - | - |
| **3 BS 2** | - | + | - | + | - | + | + | - |

Abbreviations: Activity, Negative: (-), Positive (+).

**Supplementary Table 1.** Screening of the three selected of actinobacteria for the mobilization capacity of different sources of phosphorus, cellulolytic enzymatic complex activity and Indole Acid Acetic production.
